# Potential Cost Savings for the Healthcare System by Physical Activity in Different Chronic Diseases: A Pilot Study in the Veneto Region of Italy

**DOI:** 10.3390/ijerph19127375

**Published:** 2022-06-16

**Authors:** Sara Ortolan, Daniel Neunhaeuserer, Giulia Quinto, Barbara Barra, Anna Centanini, Francesca Battista, Marco Vecchiato, Valentina De Marchi, Martina Celidoni, Vincenzo Rebba, Andrea Ermolao

**Affiliations:** 1 Sports and Exercise Medicine Division, Department of Medicine, University of Padova, Via Giustiniani 2, 35128 Padova, Italy; sara.ortolan@aopd.veneto.it (S.O.); giulia.quinto9@gmail.com (G.Q.); barbara.barra2408@gmail.com (B.B.); centanini.anna@gmail.com (A.C.); francesca.battista@unipd.it (F.B.); marcovecchiato.md@gmail.com (M.V.); andrea.ermolao@unipd.it (A.E.); 2 Clinical Network of Sports and Exercise Medicine of the Veneto Region, Via Giustiniani 2, 35128 Padova, Italy; 3 Department of Economics and Management “Marco Fanno”, University of Padova, Via Bassi 1, 35131 Padova, Italy; valentina.demarchi@unipd.it (V.D.M.); martina.celidoni@unipd.it (M.C.); vincenzo.rebba@unipd.it (V.R.)

**Keywords:** health economics, economic impact, cost-effectiveness, physical exercise, exercise training

## Abstract

Background: Sedentary behaviour (SB) and physical inactivity (PI) are associated with an increased risk of chronic diseases and a significant economic burden. This pilot study aims to estimate the possible cost savings for the Veneto Regional Health Service (Italy) due to a population-based physical activity (PA) intervention. Methods: The PA-related cost-savings were assessed for four chronic diseases in the whole and sedentary populations of the Veneto region. The SB and PA epidemiological data, regarding an additional percutaneous coronary intervention in coronary artery disease, hospitalizations in chronic obstructive pulmonary disease, surgery for colorectal cancer, and femur fracture, were obtained from national and regional administrative sources. A relative risk reduction, due to PA, was obtained from the recent literature. The annual healthcare costs were estimated using the regional diagnosis-related group tariffs. Results: The annual estimated cost-savings for the regional healthcare service related to these four outcomes: an amount between EUR 5,310,179 (if a conservative analysis was performed) and EUR 17,411,317. Conclusion: By a downward estimate, regarding the direct healthcare costs, PA interventions could lead to important cost-savings in the Veneto region. The savings would be greater when considering the cross-sectional impact on other healthcare costs, comorbidities, and indirect costs.

## 1. Introduction

Sedentary behaviour (SB) has been defined as any waking behaviour characterised by an energy expenditure of ≤1.5 metabolic equivalents of a task, such as sitting [1]. Conversely, physical inactivity (PI) is used to define an insufficient amount of moderate-to-vigorous physical activity (PA), thus not meeting the levels recommended by current guidelines. This means that a physically inactive person may not be completely sedentary and vice versa [2].

It has been shown that SB leads to an increased risk of mortality (+54%), from all causes, including cardiovascular diseases and other numerous non-communicable diseases [3,4,5,6,7,8]. Moreover, SB is associated with an increased risk of musculoskeletal diseases, sarcopenia, and a risk of falling [9,10,11]. In addition, and similarly to smoking and obesity, sitting for more than 8 h/day and not being physically active increases the mortality risk by 58% [4]. Not meeting the PA recommendations has indeed been estimated to be responsible for more than 5 million annual deaths globally [6]. Not only does SB and PI increase the risk of mortality and morbidity when it is considered individually, but the association between the two appears to confer the greatest risk [3]. In view of these facts, adults are recommended to reduce their overall SB and increase their PA [8,12]: 60–75 min of moderate-intensity exercise per day seems to eliminate the increased mortality risk associated with a long total sitting time [3,4]. However, reducing sitting time alone may be insufficient to achieve optimal health benefits; thus, it is necessary to replace sitting with vigorous walking and/or exercise, although it might be difficult to provide quantitative recommendations [7,13].

Despite the strong evidence regarding this “global pandemic”, a high percentage of the worldwide population is still sedentary, physically inactive, or both, which must be addressed by a “global action plan” [14,15].

In fact, the beneficial effects of PA on cardiovascular diseases and overall mortality have been widely investigated and documented [16,17,18,19], which may already occur at a low volume and intensity of exercise training [16]. Indeed, Myers et al. found that in both the healthy subjects and patients with cardiovascular diseases, their exercise capacity was the main predictor of overall mortality, and was stronger than other known risk factors, such as arterial hypertension, smoking, and diabetes [16]. The most recent recommendations state that adults should engage in at least 150 min to 300 min per week of moderate-intensity exercise or 75 min to 150 min per week of vigorous-intensity endurance exercise [20].

Therefore, in terms of the prevention and treatment of many chronic conditions, a tailored exercise prescription and programs, based on specific guidelines, are necessary, although still underutilised [21,22,23,24,25,26,27,28,29,30]. In order to improve this practice, awareness and knowledge must also be increased, particularly for policy makers.

From this perspective, it seems important to highlight that the PI “global pandemic” causes not only morbidity and mortality but also a major economic burden worldwide [31]. A broader implementation of exercise interventions may also lead to economic benefits [32], and numerous studies have investigated the possible cost-savings and effectiveness of PA, both in the general population and in specific non-communicable diseases [33,34,35,36,37,38,39].

Ding et al. analysed the worldwide direct healthcare costs, productivity losses, and disability-adjusted life-years (DALYs) due to PI in five major non-communicable diseases (coronary artery disease, stroke, type 2 diabetes, breast and colon cancer): the annual global healthcare costs associated with PI ranged from an average of USD 67.5 to USD 145.2 billion. In Italy, the authors estimated the direct and indirect costs attributable to PI reaching USD 1,404,701,000 in 2013, of which 78% of the direct costs were paid by the public sector [31].

On the other hand, cost-effectiveness analyses of PA interventions are much more challenging than the evaluation of PI-related costs, due to the methodological heterogeneity in the application of economic analyses in the healthcare setting and a general lack of studies on population-based PA interventions [33]. Considering the latest literature, a recent review concluded that only some interventions can be considered cost-effective; however, more research is needed on PA interventions at a sustainable cost in the primary care setting [33].

Another aspect to consider is that the cost savings vary considerably across countries, because of the differences in the healthcare systems. While most of the studies have been based on a US context, little is known about the cost-effectiveness and cost-savings regarding PA and exercise in a national health system such as the Italian one, which is almost entirely funded by the public system [35]. Based on national epidemiological data for different non-communicable diseases, in 2018, the Italian Higher Institute of Health estimated that an increase in PA would result in a reduction in the annual public healthcare costs by almost EUR 2,331,669,947, suggesting that an exercise prescription might be an interesting path to reduce public expenditure on the health system [40]. However, these data do not consider the inter-regional differences within the Italian territory, in terms of the healthcare costs and organisation. In fact, since the 1980s, the healthcare system has become increasingly regionalized and, currently, each Italian region has its own healthcare service, partially independent from the national system.

Therefore, this pilot study aims to provide preliminary economic evaluations of the PA interventions in the Veneto region (4,869,830 inhabitants, National Institute of Statistics, 2021). This Italian region has been selected because it ranks first in terms of the quality of the healthcare services provided, according to a recent analysis by the Italian Ministry of Health [41,42]. Consequently, based on the regional data, cost-saving analyses for several high-impact non-communicable diseases are provided, assessing the potential impact of a population-based PA intervention on the Veneto healthcare service. The final objective of this pilot study is to sensitise, through economic data, regional and potentially national policy makers about the health and economic benefits of promoting physical activity interventions in the population.

## 2. Materials and Methods

The present study was designed as a literature-based pilot study to evaluate the cost-saving potential of meeting the PA recommendations in different non-communicable diseases in the Veneto region, Italy.

Four non-communicable diseases of a high health impact, and in which PA is a known prognostic marker, were evaluated, i.e., coronary artery disease (CAD), chronic obstructive pulmonary disease (COPD), colorectal cancer, and femur fracture. One specific measurable endpoint for each disease was chosen to calculate the potential cost- savings that are related to PA. Furthermore, the risk of overlap with other diseases, data availability, and issues regarding analyses, were also considered when choosing the study’s endpoints.

**Figure 1:**
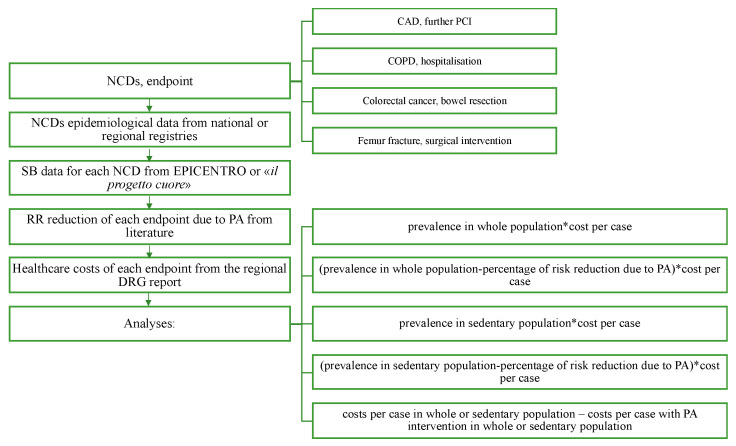
Study design. NCDs: non-communicable diseases, CAD: coronary artery disease, PCI: percutaneous coronary intervention, COPD: chronic obstructive pulmonary disease, SB: sedentary behaviour, EPICENTRO: epidemiology website for public health, RR: relative risk, PA: physical activity, DRG: diagnosis-related groups; *: multiplication sign.

To estimate the regional cost-savings that could be achieved due to PA, we proceeded with a step-wise approach, as shown in Figure 1.

Figure 1 shows the flowchart that was followed to collect and analyse the data. One measurable endpoint for each disease was chosen: further percutaneous coronary intervention (PCI) for CAD (the second intervention in patients with a previous PCI), hospitalisations for COPD, bowel resection for colorectal cancer, and surgery for a femur fracture.

The epidemiological data regarding each disease were collected from the national administrative sources of the Italian National Institute of Statistics [42] and other databases specific to each disease [43,44,45]. In particular, the performance indicators of the cardiac catheter laboratories [43], the regional registry of cancer diseases [41], and the epidemiological data system of the Veneto region were used as data sources [45]. The most recent available data was included in this evaluation. With regard to a PCI, a period of 3 years was considered due to the registry characteristics of the specific database [43].

The SB and PA data were obtained from databases of the epidemiology website for public health (EPICENTRO) as well as from the other previous projects evaluating these issues that were related to the prevention of cardiovascular diseases [46,47,48].

For each disease and study endpoint, the relative risk reduction due to PA was derived from the existing studies, preferentially systematic reviews, or a meta-analysis, if available [49,50,51,52].

The direct healthcare costs of a further PCI, hospitalisations, bowel resection, and orthopaedic surgery were evaluated for CAD, COPD, colorectal cancer, and femur fracture, respectively, utilising data on the diagnosis-related groups’ costs (DRGs) from the regional reports. All costs were expressed in euros. For each endpoint, the healthcare costs were calculated, as shown in Figure 1.

Since the PA level was unknown in the specific population, the potential cost-savings related to PA were evaluated for two possible scenarios: a broad scenario, considering the whole population affected by the specific disease, and a narrow scenario, considering the respective sedentary/physically inactive population only.

## 3. Results

### 3.1. Coronary Artery Disease

In the Veneto region, in the three-year period from 2016–2018, a total of 10,855 subjects underwent a PCI [43]. According to the epidemiological data presented in the project “Il Progetto Cuore” [46], 31% (95% IC 29.6–32.5) of the males and 41.8% (95% IC 40.2–43.3) of the females were physically inactive. Thus, to be as conservative as possible, 31% of the patients were considered for the analysis of the inactive population, leading to a total of 3365 inactive patients being treated with a PCI. Based on the data from the Italian ETICA trial, the risk of further revascularisation with a PCI was 10% in the trained patients and 27% in a physically inactive control group (RR 0.78, 95% CI: 0.66–0.89; *p* = 0.03) [49]. According to the regional DRG data, the cost of a PCI without stenting was EUR 5592.57 per patient.

In the broad scenario, considering the whole population, 2931 patients were estimated as being at risk of further revascularisation with a PCI related to the status of PI, determining a total healthcare cost of EUR 16,390,983.78. However, with a PA intervention, patients at risk for a further PCI were estimated to be 1086, for a total cost of EUR 6,070,734.74. Therefore, the possible regional cost-savings that would result from the practice of PA could amount to EUR 10,320,249.05 during a three-year period, resulting in an average annual saving of EUR 3,440,083.02.

**Figure 2:**
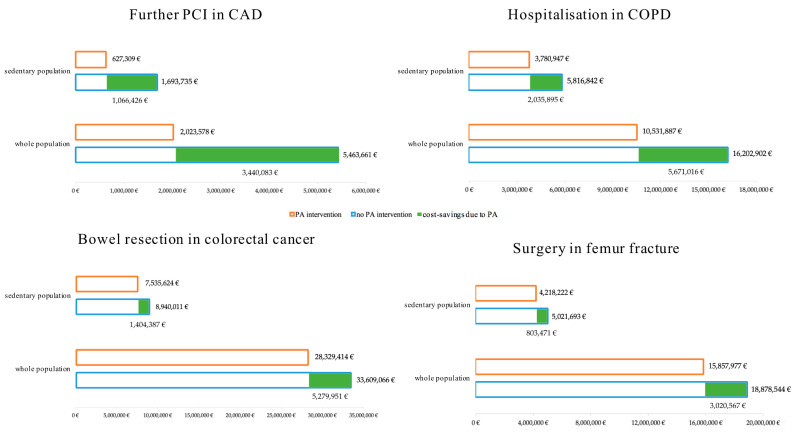
Healthcare costs and potential cost-savings in different chronic diseases. CAD: coronary artery disease, PCI: percutaneous coronary interventions, COPD: chronic obstructive pulmonary disease, PA: physical activity.

In the narrow scenario, considering only the physically inactive population, without providing a PA intervention, 909 patients would probably need further revascularisation, at a total PCI cost of EUR 5,081,204.97, while with regular PA, patients at risk for a second PCI were 337, leading to a total amount of EUR 1,881,927.77. Thus, the possible regional cost-savings that would result from the practice of PA could amount to EUR 3,199,277.21 in three years, resulting in an average annual saving of EUR 1,066,425.74 (Figure 2).

### 3.2. Chronic Obstructive Pulmonary Disease

Based on the prevalence data from the Italian National Institute of Statistics, 2018,292,000 patients were affected by COPD in the Veneto region [42]. Moreover, 35.9% of the patients affected by chronic respiratory diseases were considered sedentary [47]. In 2015, 6685 hospitalisations had COPD as a primary admission diagnosis [53], of which 2400 were calculated to occur in sedentary patients. In a 20-year follow-up cohort study by Garcia-Aymerich et al., regular PA reduced the risk of hospitalisations in patients with COPD by 30–40% (RR: 0.72, 95% CI 0.53–0.97, *p* = 0.03) [50]. According to the regional DRG tariffs, the cost of each hospital admission for COPD was EUR 2423.77 per patient.

In the broad scenario, for the 6685 hospitalised patients, the total healthcare costs for hospital admissions were EUR 16,202,902.45 per year, which could be reduced with regular PA to an average of EUR 10,531,886.59. Indeed, the possible cost-savings that would result from the practice of PA by reducing patients’ hospitalisations could amount to EUR 5,671,015.86 per year.

In the narrow scenario, considering only the sedentary patients without a PA intervention, the estimated annual healthcare cost for hospitalisations would amount to EUR 5,816,841.98, while a PA intervention would reduce this average cost to EUR 3,780,947.29 per year. Thus, the possible regional cost-savings that would result from the practice of regular PA could yearly amount to EUR 2,035,894.69 (Figure 2).

### 3.3. Colorectal Cancer

In 2015, 3602 patients were affected by colorectal cancer in the Veneto region [44], and a total of 3318 colorectal resections were performed as medical treatment. Moreover, 2919 surgeries for the colon and 399 for the rectum bowel were registered, with or without surgical complications [53]. Since the age at an increased risk of colorectal cancer is 50–70 years, 26.6% of this population was associated with SB [48], leading to an estimated total of 883 sedentary patients per year being diagnosed and treated for colorectal cancer in Veneto. Based on a large cohort study published by Moore et al., high levels of PA are associated with a 16% and 13% lower risk of colon (HR = 0.84, 95% CI 0.77–0.91, *p* < 0.001) and rectal cancer (HR = 0.87, 95% CI 0.80–0.95, *p* = 0.001), respectively [51]. According to the DRG data, the costs of a colon resection were EUR 13,729.72 or EUR 6716.05 per patient, depending on the potential post-surgical complications; costs of a rectum resection were EUR 10,767.76 when the complications had to be treated, and EUR 6141.14 for those without any complications.

In the broad scenario, the total healthcare costs for 3318 bowel resections (with or without post-surgical complications) were EUR 33,609,065.75, while a PA intervention might reduce the estimated associated healthcare costs to EUR 28,329,414.43 due to a reduction in disease incidence. The possible cost-savings that would result from the practice of PA could thus amount to EUR 5,279,651.32 each year.

In the narrow scenario, considering that only the sedentary patients are estimated to undergo bowel resection due to cancer, no PA would result in the EUR 8,940,011.49 of specific surgery costs, while a PA intervention would reduce the annual costs to EUR 7,535,624.24. The possible regional cost-savings that would result from the practice of PA could, thereby, amount to a yearly EUR 1,404,387.25 (Figure 2).

### 3.4. Femur Fracture

In 2010, 5848 patients had femur fractures in the Veneto region, and 93.1% of them underwent surgery (52% for total prosthetic surgery, 27% for partial prosthetic surgery, 14% for osteosynthesis) [45]. Since elderly people are at a greater risk of femur fracture, 26.6% of SB was considered [48] for an estimated 1556 sedentary patients with femur fractures each year. A recent systematic review and meta-analysis found that long-term exercise decreased the risk of fractures by 16% (RR: 0.84, 95% CI 0.70–1.00, *p* = 0.047) [52]. According to the regional DRG data, the costs for femur fracture surgery were EUR 3467.46 per patient.

Thus, in the broad scenario, the total healthcare costs for 5444 surgery interventions were EUR 18,878,544.36, while regular PA might reduce these costs to EUR 15,857,977.26 due to a preventive risk reduction. The possible regional cost-savings that would result from the practice of PA could thereby amount to EUR 3,020,567.10 per year.

In the narrow scenario, considering only the 1448 sedentary patients that were estimated to undergo femur fracture surgery, without PA intervention, EUR 5,021,692.80 of specific healthcare costs are needed, while a PA intervention would lead to a reduction of EUR 4,218,221.95 of surgery-related costs. Thus, the possible regional cost-savings due to the practice of regular PA could amount to EUR 803,470.85 each year (Figure 2).

Figure 2 shows the healthcare costs of the specific endpoints of the four chronic diseases, considering the impact of a PA intervention. In green, the resulting potential annual cost-savings due to PA for each disease are shown. The shown data are expressed in euros.

## 4. Discussion

The underlying aim of this study was to investigate the potential cost-savings of a PA intervention for a specific regional healthcare service in Veneto, Italy. Four non-communicable diseases with a high impact on the burden of the public health system were evaluated, regarding one specific measurable endpoint. Based on the analyses performed, it has been shown that, even just by considering one specific endpoint for just the four non-communicable diseases investigated, the healthcare service of the Veneto region could save EUR 17,411,317.29 or EUR 5,310,178.53 per year, when considering the whole or sedentary population, respectively.

In view of the increasing budget constraints that policy makers have to face, both at the national and regional levels, which are likely to be exacerbated after the current pandemic, the results from this study should raise the interest of policy makers. According to the presented data, they should seriously consider investing in further studies and in the promotion as well as the implementation of PA and exercise in public health and healthcare settings. These results are even more interesting when considering the conservative nature of our analyses. Indeed, this cost-saving evaluation purposefully neither takes into account the possible cost-savings related to the benefits of PA on other disease-related outcomes, nor the pleiotropic beneficial effects on the associated comorbidities and different non-communicable diseases. In the 1980s, Dr. Robert Butler, the founder of the National Institute on Aging in the US, stated that “if exercise could be packaged in a pill, it would be the single most widely prescribed and beneficial medicine in the nation”. Nowadays, we know how correct that statement is, not only because of the beneficial effects of PA on health and disease [7,54,55,56], but also due to the global economic burden of PI, which must be specifically addressed by public health initiatives [31,37,39,57]. Moreover, it is noteworthy that all the cost-effectiveness analyses on the PA interventions focus on specific measurable outcomes and/or specific non-communicable diseases, which leads to an underestimation of the real impact of the “Exercise Pill”. Indeed, it is rather impossible to quantify all the benefits of PA on the primary, secondary, and tertiary preventions of non-communicable diseases.

Furthermore, the results that are reported have to also be considered conservative for other reasons. In the present study, the estimated healthcare costs and cost-savings were provided both for the whole disease-specific population and for the disease-related sedentary sub-group. This approach was applied to provide the most conservative evaluation possible, leading to a range of estimated cost-savings. It is also known that the starting level of PA is directly related to possible health benefits. The less active a subject is prior to a PA intervention, the greater the health gains will be when some PA is added [2,33]. Consequently, it can also be assumed that in the sedentary population, the cost-effectiveness and cost-savings of the PA interventions are superior.

Moreover, the nationwide proportion of the elderly population regularly engaging in some PA is even lower, reaching only 9.2% among women and 11.2% among men [58]. In that view, the more conservative evaluation presented above may considerably underestimate the true cost-saving potential of PA in the non-communicable diseases considered, which are of higher prevalence in the elderly population.

In addition, in 2019, the physically inactive population in the Veneto region was 23.2%, compared with a national average of 35.6% and a southern Italian average of 47.8%. During the SARS-CoV-2 pandemic, these inter-regional differences became even more pronounced: in 2020, the physically inactive population was 21.3% and 49.3% in the Veneto region and in Southern Italy, respectively. Therefore, it seems reasonable that the economic burden of PI and the potential cost-savings of the PA interventions throughout Italy may be even greater than in the investigated region alone [30,59].

Misperception must also be considered when discussing SB and PA levels, which are usually self-assessed and often under- or over-estimated, respectively [59]. These findings, particularly regarding the underestimation of SB, are in line with the hypothesis that the present evaluation probably undervalues the direct healthcare cost-savings. In addition, this study—and many previously published cost-effectiveness analyses—investigated only the health-oriented perspective, hence, they did not consider the broader benefits of productivity that resulted from avoiding complications related to SB and PI [35].

The known PA-associated health benefits and possible cost-savings reported in this study call for focused national health system initiatives, and also a general greater effort in the implementation of exercise prescription in healthcare settings. In support of this statement, an evaluation conducted by the Italian Higher Institute of Health in 2018 estimated that the direct healthcare costs attributable to physical inactivity for four chronic diseases (i.e., breast and colon cancer, coronary disease, type 2 diabetes) amounted to a total of EUR 1561 million. In this context, a 5% reduction of PI could result in a saving of EUR 78.5 million, while a 20% reduction would save about EUR 312.7 million in direct healthcare costs [40]. Although the diseases considered and the methodological approach are different from those in our study, these national data further highlight the entity of possible economic gains from implementing physical activity in the Italian healthcare system.

The World Health Organization, in 2018, developed a plan to increase the global levels of PA, and the target was a 15% relative reduction in the global prevalence of PI by 2030 [60,61]. Initiatives to increase the level of PA also exist at a European level, such as the European Physical Activity on Prescription project, which is based on the Swedish evidence-based model and has recently been recognised as a best practice [62,63,64]. Italy and other countries are also involved in another global and European initiative called Exercise is Medicine^®^, which so far, has been introduced in about 37 countries around the world [65]. The national, regional, and local policy makers should engage more in these types of initiatives in order to achieve the highest benefits from the promotion of PA. A recently published cost-effectiveness analysis suggests that by investing in careful urban design and promoting PA, a significant impact on public health can be obtained without the need to mobilise substantial resources from the healthcare budget in Italy [35]. Moreover, such implementation strategies would also lead to a reduction in the morbidity burden, for example, by delaying or preventing the onset of chronic diseases [35]. Indeed, this would, even more, affect the national impact on health economics in the long-term.

All the above-mentioned evidence-based initiatives call for a paradigm shift in the society and healthcare system as well as strong support by governance and policy makers. Additional efforts on the implementation of these highly effective treatment options are thus desperately needed, which can be achieved in part by enhancing the knowledge and understanding of the multiple benefits of PA, especially on the economic burdens. To achieve this goal, governments and policy makers should pave the way for societies in which PA is a socioeconomic foundation of a healthy, enjoyable, safe, affordable, and convenient lifestyle and where exercise prescription is effectively implemented as a treatment modality in healthcare systems.

Since the aim of this study was not to provide exact data on the cost savings but rather to estimate the potential impact of a PA intervention on the economic burden of the healthcare system in a real-world setting, this retrospective analysis comes with some intrinsic limitations. Firstly, data of the evaluated non-communicable diseases were extracted from different registries in different years. The measurable economic outcomes are not readily accessible for all chronic diseases, which forced us to focus only on four, representing high-impact pathologies. Furthermore, using the DRGs’ costs to analyse the cost savings, only one aspect of the healthcare costs has been considered, evaluating just one specific endpoint. Moreover, the current COVID-19 pandemic increased SB and PI in the general population, leading to a further negative impact on healthcare costs [66].

Hence, based on the outcomes of this pilot study, a prospective and controlled cost-saving analysis with comprehensive data from the Veneto region should be designed. The specific economic impact of increased PA on the regional healthcare service could be evaluated for different settings and diseases [33,38,39], thus, applying it also at a national level. However, it may be of interest to also consider the costs of PA promotion and its specific interventions in order to provide a more accurate estimation of the potential cost-effectiveness of the different PA interventions. Finally, the indirect costs related to productivity should also be specifically addressed by future research projects.

## 5. Conclusions

PA interventions were shown to be cost-saving in the Veneto region if the direct healthcare costs are analysed, and it can be assumed that the impact is largely underestimated, particularly when considering the pleiotropic effects and indirect costs. Regional and national policy makers should, thus, seriously consider investing in PA promotion as well as in implementation strategies for exercise prescription in public health services and healthcare settings in order to improve the health status of citizens and to save public money.

## Data Availability

The data that support the findings of this study are available from the corresponding author upon reasonable request.
